# Measurement of Wheel Skidding on Racing Wheelchairs †

**DOI:** 10.3390/mps8020028

**Published:** 2025-03-06

**Authors:** Nolwenn Poquerusse, Arnaud Hays, Aurélie Cortial, Opale Vigié, Ilona Alberca, Mathieu Deves, Lorian Honnorat, Safiya Noury, Bruno Watier, Arnaud Faupin

**Affiliations:** 1 Jeunesse Activité Physique Sport Santé Laboratory, University of Toulon, 83130 La Garde, France; aurelie.cortial@univ-tln.fr (A.C.); opale.vigie@univ-tln.fr (O.V.); alberca@univ-tln.fr (I.A.); mdeves@fft.fr (M.D.); lorian.honnorat@univ-tln.fr (L.H.); noury@univ-tln.fr (S.N.); 2 Health Improvement Through Physical Exercise Human Laboratory, Aix Marseille University, 13002 Marseille, France; arnaud.hays@univ-amu.fr; 3 Laboratoire d’Analyses et d’Architecture des Systèmes, Université de Toulouse, CNRS, 31031 Toulouse, France; bruno.watier@laas.fr

**Keywords:** wheelchair racing, biomechanics, skidding

## Abstract

In the context of wheelchair racing, research primarily focuses on studying wheelchair ergonomics and determining kinematic, kinetic, and rolling resistance variables. One factor identified as influencing athletes’ performance is wheel skidding on the ground, a parameter complementary to rolling resistance. The objective of this study, therefore, is to identify, within a laboratory setting, the parameters that influence the risk of skidding in racing wheelchairs by measuring skidding torque. The ultimate goal is to enhance athletes’ performance by optimizing the interaction between the athlete and their wheelchair, and the wheelchair and the environment. In this perspective, four parameters were examined: the type of tubular, the camber angle, the tire pressure, and the load applied to the wheel using a skidometer. This tool characterizes a tire’s grip on a surface by measuring torques. The aim is to develop a system for classifying tire grip on dry athletics track at ambient temperature. The findings revealed that only the effects of load and tubular type had a significant impact on the torque values obtained. The tire that minimized the risk of skidding, among all tested combinations, is the Vittoria Pista Speed 23–28″. Furthermore, as the mass applied to the wheel increases, so do the resulting torques. This implies that a heavier athlete would require a greater force to be applied to the hand rim for the tire to skid. However, it was also demonstrated that the risk of skidding in a racing wheelchair is unlikely, as the torques obtained were over a range of 90 to 190 Nm. These values far exceed those typically exerted by para-athletes, which are a maximum of 60 Nm. The long-term goal would be to adjust the mode of torque application on the wheel using the skidometer for a more realistic field approach.

## 1. Introduction

Pushing capacity is a key parameter in racing wheelchair performance. One way to improve racing wheelchair speed is to increase energy transfer to the wheelchair. Several variables may affect energy transfer, from equipment design, athlete anthropometry, pathology and neuromuscular capabilities, to the specific motor control strategy chosen to accomplish the task [1,2,3]. The pushing technique is a crucial factor in athlete performance [4] as it ensures effective transmission of forces applied from the hand rim to the surface, thereby minimizing energy dispersions. Indeed, the forces exerted on the hand rim of the wheelchair must be optimized to enable efficient torque transmission to the surface [5]. In addition to pushing parameters, rolling resistance [6,7,8] and the coefficient of friction [9,10,11] are complementary parameters considered. The latter indicates the grip of one surface relative to another [12], here between a tire and a ground surface.

In the context of this study, the goal is to determine the parameters, among four selected, that influence the risk of skidding, by measuring the torque applied to the wheel that leads to its skidding. Ivanov et al. [13] conducted a study to determine the skidding grip torque of a tire. Following this method, this study introduces a new approach capable of measuring tire grip through the calculation of torques (referred to as grip loss and recovery torques). The protocol focuses solely on measuring circular rotation torques with a non-fixed wheel [13], isolating the wheel from the rest of the wheelchair. This new protocol is tested on a tool called a skidometer. Specific parameters are studied to understand their influence on the determined torques, namely, tire pressure, wheel camber angle, and the load applied to the wheel. The selection of these parameters was based on recommendations from the scientific literature [10,11,13]. The values of the variables and the types of tubulars tested were selected by the athletics federation prior to the experimentation. Based on the existing literature, each parameter studied is expected to influence skidding differently. Tire pressure plays a key role in ground adhesion: higher pressure reduces the contact patch, potentially decreasing grip and increasing the risk of skidding [10,13]. Conversely, lower pressure can improve grip by increasing tubular deformation but may also lead to higher rolling resistance. Wheel camber angle affects force distribution on the ground; a positive camber generally enhances lateral stability by increasing the contact area, while a neutral or negative camber may reduce stability and facilitate skidding [11]. Finally, the load applied to the wheel directly impacts the normal force on the ground: a higher load increases this force, which tends to enhance grip and require a higher torque to initiate skidding [13]. This study aims to experimentally assess these influences using the proposed protocol. Indeed, the hypothesis, based on the results of previous work and the objectives of our research, is as follows:

Hyp: The application of the proposed method will enable the identification of parameters among the camber angle, pressure, the load applied to the wheel, and tubular type that influence the risk of wheel skidding in wheelchair racing, by the measure of grip recovery and loss torque.

This knowledge could serve as a foundation for a more precise optimization of the individual settings of sports wheelchairs.

## 2. Protocol and Method

### 2.1. Material and Method

This study is part of the PARAPERF project, registered under the “France 2030” program (reference ANR-19-STHP-0005). To achieve this research, a skidometer was developed by the HIPE Human Lab at Aix-Marseille University to measure the skidding torque of a wheelchair’s rear wheel.

**Figure 1:**
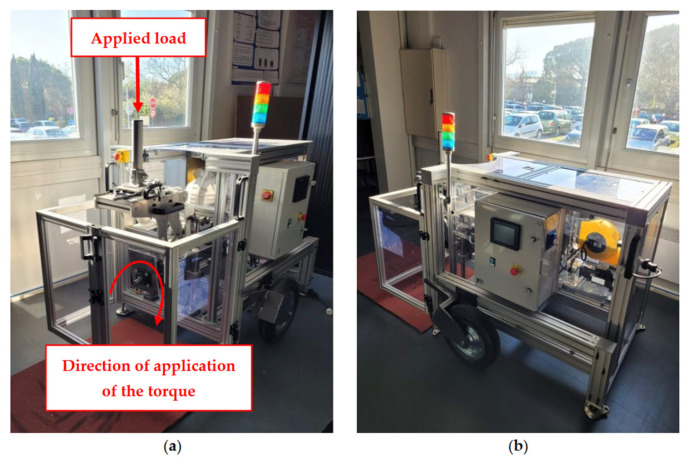
Skidometer with (**a**) front view, (**b**) rear view (electronics and motor).

The tool operates as follows: a motor is mounted on the axle of a wheel via a reducer. The motor applies a gradually increasing torque, at a constant rate, until the point of skidding of the tire on the athletics track is reached. The skidometer has an acquisition frequency of 2 Hz. The measurement bench is also equipped with a tube for loading the system with calibrated weights (Figure 1a). This tube is mounted on a rail where a light beam indicates the location where the weights are applied to the wheel. The load represents the mass of the athlete distributed over a single wheel (approximated as the athlete’s mass divided by 2) and is, therefore, positioned at the center of the tire. It is assumed here that the athlete’s weight is uniformly distributed across both wheels of the wheelchair. Indeed, the modeling of the athlete’s weight was performed in the plane of the wheels, which means that the additional torque generated by the athlete’s weight, situated in the plane of symmetry of the wheelchair, was not considered. This choice is justified by the need to isolate the wheel for analysis, making it impossible to consider this torque in our configuration. This modifies the actual weight distribution, which can affect stability and balance calculations, as well as the forces and torques acting on the wheel. The dynamic behavior of the wheel, its wear, and durability may also be influenced, making comparisons with real data less precise. However, this approach simplifies the analysis and allows for a focus on the intrinsic characteristics of the wheel itself. It also provides a basis for comparison for future studies where the additional torque could be integrated, enabling a better understanding of the individual contributions of various factors.

**Figure 2:**
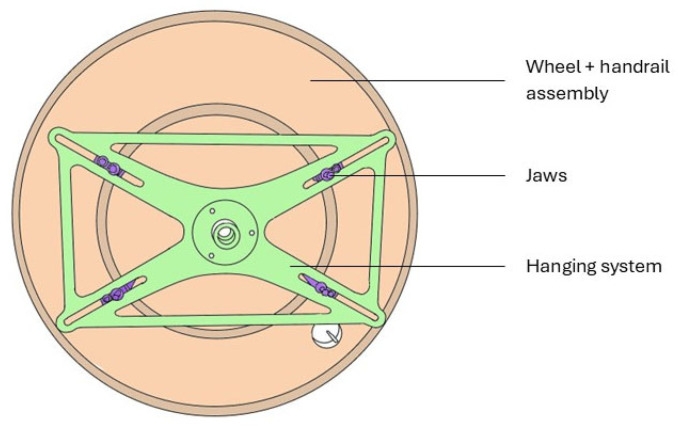
Wheel mounting system on the handrail.

Moreover, a pneumatic actuator allows for the adjustment of the camber angle of the wheel. A transducer, located at the wheel axis, indicates the value of the camber angle. The skidometer is equipped with a touchscreen to visualize in real-time the values of torques applied to the wheel. The wheel is mounted on the skidometer using a support fixed to the hub. The support is adjustable to accommodate wheels of different sizes. The entire system is divided into three parts: a mounting system (support + hub), the jaws, and a wheel assembly with the handrail (Figure 2). The tests were conducted on a dry Mondo athletic track (MondoTrack WS 13.5 mm).

At the start of the test, the wheel is stationary. When the skidding torque is reached, this initiates the rotation of the wheel, as this corresponds to the moment when the tire skids from the surface. The grip recovery torque corresponds to the moment when the tire re-establishes contact with the ground. This value is determined by averaging the last 10 recorded data points before the test stops, corresponding to a 5 s average. The wheelchair parameter study was conducted on a dry Mondo athletics track at ambient temperature (20 °C). The experiments were performed with unheated tires. Only the rear wheel tire of the racing wheelchair is analyzed.

To assess the influence of various parameters of the racing wheelchair on the measured torques, this study focused on the impact of the type of tubulars used, the wheel camber angle, tire pressure, and the load applied to the wheel. The values of the variables and the types of tires tested were selected by the athletics federation prior to the experimentation and are listed in Table 1. Tire pressures were set using a manual pump equipped with a pressure gauge accurate to ±0.1 bars. To prevent track wear, the testing area was moved to a new section every three trials.

### 2.2. Experimental Design

Conducting tests for all possible combinations of each factor studied would have required 288 trials ($n_{trial}=n_{tire}\cdot n_{pressure}\cdot n_{load}\cdot n_{camber}\cdot 2_{repetition}$). Since the experiment is time-consuming (averaging 3 min per trial), it was necessary to implement an experimental design. The Taguchi method [14] employs specific experimental designs to identify the most influential factors. This method enables the creation, based on the number of factors and their levels, of an optimized table of trials, including only the necessary and sufficient trials for a comprehensive analysis. This allows for the minimization of the number of interactions to be tested while maintaining the rigor and quality of the study. Indeed, orthogonal arrays are created to simultaneously test multiple factors, thereby reducing the total number of experiments required. To create this table, factor interactions were not taken into account. The objective was to determine the effect of each factor individually and independently, identifying the isolated contributions of each parameter to the risk of wheel skidding in wheelchair racing. This methodological choice simplifies the analysis and enables targeted optimization of each parameter. However, it has limitations. Specifically, it overlooks potential interactions between factors, which may have a substantial impact on the overall wheelchair dynamics. This methodological compromise reflects a pragmatic approach for preliminary optimization while highlighting the need for further studies to refine practical recommendations. Since the six levels of the factor $n_{tire}$ were not considered due to the lack of suitable tables with six levels for this study, the L16 ($2^{3}4^{4}$) table was chosen as a model [15] (Table 2). This results in a total of 96 combinations to test ($n_{comb}$ $=16\;\cdot \;6_{tire})$, with each condition being repeated twice ($n_{tot}=192$). It was estimated that by using the Taguchi method, the experimentation time was reduced by 5 h, corresponding to a time saving of 34.7%, without compromising the quality of the analysis. All manipulations were completed in one day. The choice of the L16 table varies according to the number of factors studied. Thus, the selection of the appropriate Taguchi table is made by calculating the degrees of freedom [16].

### 2.3. Statistical Method

**Figure 3:**
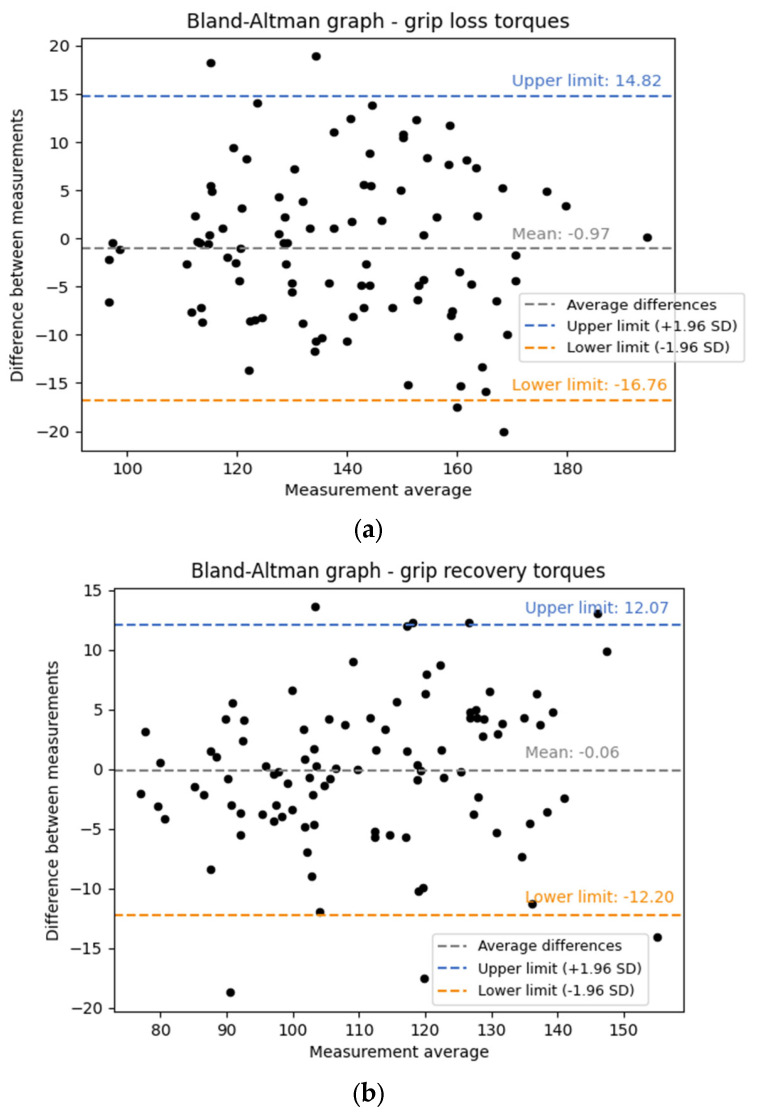
Bland–Altman plot for (**a**) grip loss torques, (**b**) grip recovery torques.

To ensure the reliability of our findings, Bland–Altman tests were conducted to evaluate the repeatability of the measurements (Figure 3), as each was repeated twice. This method quantifies agreement between measurements by calculating differences and limits of agreement, helping identify any systematic bias or variability. Including Bland–Altman tests validate the consistency of our measurement techniques, ensuring that conclusions are based on reliable and reproducible results. This is essential for our study’s objective of optimizing wheelchair settings to minimize skidding risk, as it ensures that measured torques accurately reflect performance under controlled conditions. Therefore, for each measured value of the torques, it was necessary to calculate the following values:

(1) Difference=Measure 1−Measure 2,

with Measure 1: measured value for a specific combination for trial 1, Measure 2: measured value for a specific combination for trial 2.

(2) $$Mean=\frac{Measure\;1+Measure\;2}{2},$$

(3) $$Limits\;of\;agreement=Mean\_diff\pm 1.96\times s\_d_{diff}$$

with: Mean_diff: the mean of the differences and $s\_d_{diff}$: the standard deviation of the differences.

Moreover, the objective of the statistical analysis is to determine whether each of the parameters studied (pressure, camber angle, load, tubular type) has a significant effect on the determination of skidding torques. Initially, the Shapiro–Wilk test showed that all measurements were normally distributed. Multiple one-way ANOVAs were then performed for each dependent variable (grip loss torques and grip recovery torques) using JASP software, version 0.19.0.0 (JASP Team, 2024). A factor effect is considered significant if the *p*-value p<0.05. These values were determined by considering the results of the two trials independently and were calculated separately for each condition.

Mauchly’s sphericity test was conducted to check if the sphericity assumption was violated; if so, a Greenhouse–Geisser correction was applied. A Bonferroni adjustment was made for multiple comparisons with p = 0.05. For each significant difference, the effect size *η*^2^ was calculated using the following equation.

(4) $$\eta^{2}=\frac{{SS}_{effect}}{{SS}_{effect}+{SS}_{error}},$$

where *η*^2^ represents the partial eta-squared of the variable under consideration; $SS_{effect}$ is the sum of squares for the effect of the variable under consideration; and $SS_{error}$ is the sum of squares for the errors of the variable under consideration. Effect size was interpreted according to Cohen [17]: small ($\eta^{2}\;=\;0.01$), medium ($\eta^{2}=\;0.06$), and large ($\eta^{2}\;=\;0.14$).

The post hoc test is conducted after the analysis of variance when p < 0.05 (the significance threshold). For each significant difference, Cohen’s effect size, denoted as d, was calculated using the following equation:

(5) $$d=\;\frac{mean(x_{0})+mean(x_{1})}{s.d(x_{0},x_{1})}$$

where $x_{0}$: 1st trial, $x_{1}$: 2nd trial, $s.d(x_{0})$: standard deviation between the two trials.

**Figure 4:**
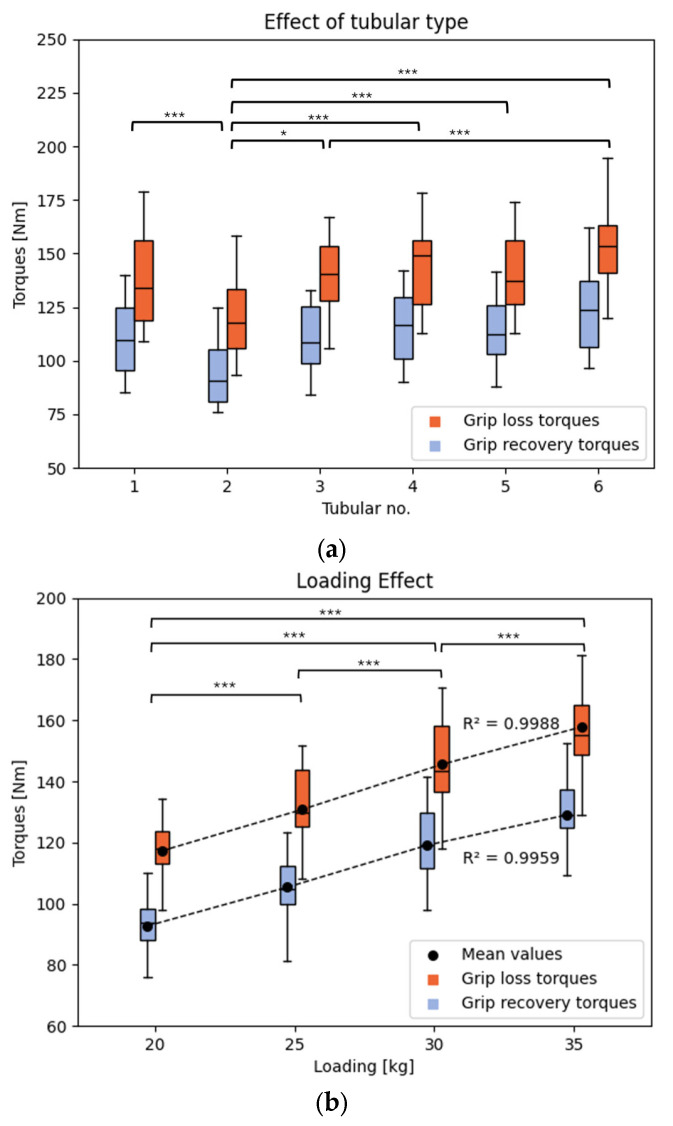
Boxplot graphs of the influence of the effects of (**a**) tubular type; (**b**) load on the resulting torques. The results of the post hoc tests are shown in this figure. Significance levels of the post hoc tests: * low (*p* < 0.05), *** high (*p* < 0.001).

Effect size was interpreted according to Cohen [17]: small (d=0.2), medium (d=0.5), and large (d=0.8). The results of the post hoc tests are directly displayed on the graphs (Figure 4) using effect sizes.

## 3. Results

### 3.1. Reliability Study

Figure 3 shows that nearly all values of grip loss torques (Figure 3a) and grip recovery torques (Figure 3b) lie within the limits of agreement. A slight systematic bias is observed, with a mean difference of −0.97 Nm for grip loss torques and −0.06 Nm for grip recovery torques, indicating a minimal systematic difference between measurements. In both graphs, the points are distributed around the mean line, with some values exceeding the limits of agreement, suggesting moderate variability. The grip recovery torque measurements appear more reliable, with slightly smaller deviations from the mean. For grip loss torques, the limits of agreement extend from −16.76 Nm to 14.82 Nm around the bias, totaling 31.58 Nm, which represents about 23% of the mean value ($\frac{31.58}{138.02}\;\approx \;0.23$). This range indicates notable but moderate variability. For grip recovery torques, the limits of agreement range from −12.20 Nm to 12.07 Nm, for a total range of 24.27 Nm, corresponding to approximately 22% of the mean ($\frac{24.27}{110.31}\;\approx \;0.22$). Thus, the relative variability is comparable to that of grip loss, though slightly narrower, confirming a marginally better agreement for grip recovery torque measurements.

### 3.2. Representation of the Effect of Each Factor on the Torques Obtained

**Figure 5:**
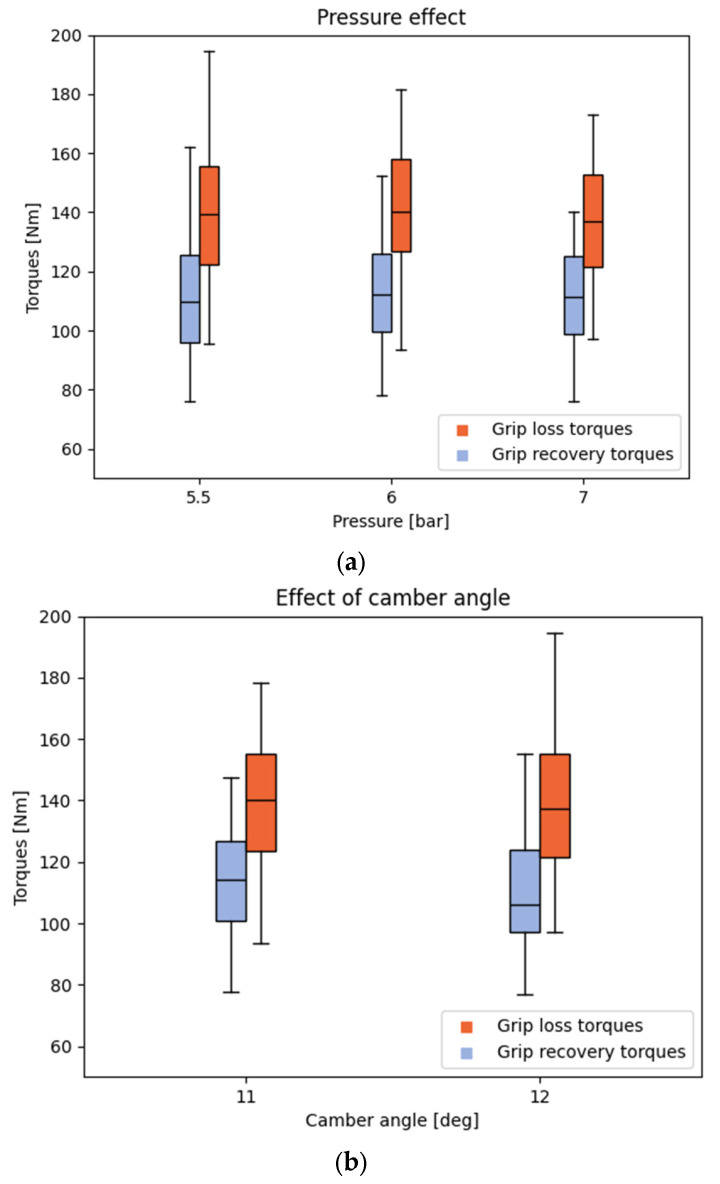
Boxplot graphs of the influence of the effects of (**a**) camber angle; (**b**) tubular pressure, on the resulting torques.

The results are presented as boxplot graphs, which graphically display the location, spread, and skewness of groups of numerical data through their quartiles. Each graph illustrates the specific effect of a parameter on the resulting torques (Figure 4 and Figure 5) and the statistics are presented in table form (Table 4). The average grip loss torque across all tires is 138 Nm ± 63 Nm, and the average grip recovery torque is 110 Nm ± 50 Nm.

Referring to Table 4, only the tubular type and the applied load on the wheel lead to a significant difference on the resulting torques (tubular effect: $p_{glt}<0.001$, $p_{grt}$ <0.001; load effect: $p_{glt}$ <0.001, $p_{grt}<0.001$, with $p_{glt}$ representing the effect of the factor on the grip loss torque and $p_{grt}$ representing the effect of the factor on the grip recovery torque). The post hoc test indicates that all applied loads have a significant influence on both grip loss and recovery torques ($p_{bonf}\leq \;0.001$). To complement this analysis, Figure 4a reveals that the effects vary depending on the tubulars used. The torque values obtained with tubular no. 2 are significantly lower than the average values. Additionally, it is noted that the highest torque values are obtained with tire no. 6. The post hoc test shows that a significant difference is observed between tubular no. 2 and all other tubulars ($p_{bonf}\leq \;0.001$ except between tires 2 and 3: $p_{bonf}=0.024$) and between tubular no. 3 and no. 6 ($p_{bonf}\leq \;0.001$). No significant difference is found between the other tubulars.

## 4. Discussion

The aim of this study was to identify the parameters between four key factors, i.e., pressure, tubular type, camber angle, and applied load on the wheel, that influence the risk of wheel skidding in racing wheelchairs. To accomplish this, the grip recovery and grip loss torques were measured using a skidometer, a tool designed to characterize a tire’s grip on a surface.

Initially, the analysis of the Bland–Altman plot indicated that the utilization of the skidometer to investigate the skidding of a wheelchair wheel on a track is a reproducible method (Figure 3). Specifically, nearly all values lie within the agreement limits and are distributed around the mean line. The variability of the measurements is moderate for both grip loss and grip recovery torques. However, the results should be interpreted with caution, as the trials were repeated only twice due to time constraints. To enhance the reliability of these findings, it would be advisable to replicate this test with a minimum of three trials for each measurement.

The hypothesis, which assumed that the proposed method would enable the identification of parameters among the camber angle, pressure, the load applied to the wheel, and tubular type that influence the risk of wheel skidding in wheelchair racing, has been partially validated. Based on the statistical studies conducted, only the load and tubular type demonstrated significant effects (Table 4) on the torques obtained. This suggests that, for the tested values, the skidding of the racing wheelchair wheel is primarily influenced by the athlete’s weight and the type of tubular used, rather than tire pressure or the camber angle of the wheel. However, a *p*-value indicates the probability of observing the data, assuming the null hypothesis is true. In this context, a significant *p*-value suggests that the load and tubular type have a genuine effect on wheel skidding, but non-significant *p*-values for tire pressure and camber angle do not necessarily mean these factors have no influence; rather, their effects may be more subtle or context-dependent.

Figure 4b illustrates the influence of the applied load on the resulting torques. The effect of loading is linear, with high coefficients of determination (R^2^ = 0.9988 for grip loss torques and R^2^ = 0.9959 for grip recovery torques). This linearity indicates that the risk of skidding is inversely proportional to the athlete’s weight; lighter athletes experience a higher risk of skidding compared to heavier athletes. This finding aligns with biomechanical principles, as a heavier athlete requires a greater force applied to the hand rim to induce tire skidding due to increased normal force and consequent frictional resistance. However, the application of Coulomb’s law is not straightforward in this context. The deformable nature of the tubular material (rubber tread with various coatings) and the heating of the tire in contact with the ground introduce non-linearities in the coefficient of friction (μ). These factors invalidate the direct application of Coulomb’s law, which assumes a constant coefficient of friction [18]. The dynamic interaction between the tire and the ground, influenced by factors such as material properties, temperature, and loading conditions, necessitates a more complex biomechanical analysis. In conclusion, it is crucial to consider the athlete’s weight when assessing the risk of wheelchair skidding. The biomechanical interplay between applied forces, normal forces, and frictional dynamics underscores the need for a comprehensive understanding of these factors to mitigate skidding risks effectively.

Figure 4a illustrates the influence of tubular type on the resulting torques. In the context of wheelchair racing, the primary goal is to minimize the risk of wheel skidding, thereby achieving high grip loss and recovery torques. Additionally, the difference between grip loss and recovery torques should be minimized, as it reflects the wheel’s recovery speed after skidding on the ground. Regarding the chosen tubular types, one tubular stands out: tubular no. 6 (Vittoria Pista Speed 23–28″). This tubular is known for its durable construction and grip and exhibits the highest grip loss torque ($t_{max}=152.8\;Nm$), while showing a grip recovery torque similar to the other tires. This combination ensures a high level of traction and quick recovery from skidding, which is crucial for maintaining control and performance during races. The specific characteristics of the Vittoria Pista Speed, such as its robust construction and optimized tread pattern, contribute to its superior performance in grip loss and recovery.

Moreover, this research found that pressure values did not have a significant effect on wheel skidding. Indeed, tire pressure influences the contact patch with the ground and, consequently, tire grip. However, this contact area also depends on other parameters such as tire type, athlete mass, speed, and environmental conditions. Therefore, analyzing pressure independently of these factors may not fully capture its role in skidding behavior. Furthermore, statistical analysis of the data revealed that, within the tested pressure range, its effect on skidding was not significant. This does not imply that tire pressure has no influence under all conditions, but rather that, under the specific experimental conditions of this study, its impact on wheel skidding did not reach statistical significance. This lack of a significant effect could be explained by the fact that, within the tested pressure range, variations in grip remain relatively small, and other parameters, such as applied load or camber angle, play a more dominant role in triggering skidding.

Additionally, the absence of a notable effect from camber angle (Figure 5b; Table 4) contradicts the scientific literature and can be explained by the range of values tested. Previous studies have evaluated the influence of the camber angle on various parameters. Ott et al. [19] demonstrated that the camber angle has a weak influence on rolling resistance. However, their study only focused on evaluating angles ranging from 0° to 5°. On the other hand, Faupin et al. [20] showed that an increase in the camber angle raises the residual torque, which leads to deceleration. Moreover, a change of 1° may not be sufficient to alter the contact area between the wheel and the ground, thus resulting in no noticeable change in force distribution. Therefore, it would be interesting to expand the range of tested angles while respecting the settings used by wheelchair athletes. This would provide a more nuanced understanding of how the camber angle affects grip loss and recovery torques, potentially leading to optimized configurations for enhanced performance in wheelchair racing.

Additional tests were conducted with the Vittoria Pista Speed 23–28″ tubular to verify the influence of pressure on grip loss and recovery torques over a wider range of values. This was performed to confirm or refute the initial results indicating that pressure has no effect on the obtained torques. The experiment was carried out by testing pressures of 6, 8, 10, and 11 bars. These values were chosen based on the product’s technical specifications to ensure a comprehensive evaluation. The results indicated that a pressure of 11 bars minimizes the risk of skidding. The effect of pressure on the obtained torques evolves in an increasing manner with the applied mass. This suggests that there is an optimal pressure range where the grip loss and recovery torques are maximized, and this range is influenced by the athlete’s weight. It appears more beneficial to increase the tire pressure when the athlete’s weight is higher. However, to confirm this initial impression, a statistical study would be necessary. Such a study would help to quantify the interaction between tire pressure, athlete weight, and the resulting torques, leading to more precise recommendations for optimizing performance in wheelchair racing.

However, the torques obtained in this experiment are below the values achieved by para-athletes. The study by Miyazaki et al. [21] determined that the tangential forces exerted by elite para-athletes on the hand rim reach a maximum of 150 N (in a propulsion test of 10 cycles at a speed of 5.56 m/s). To determine the corresponding torque, the following mathematical relationship is applied:

(6) $$T_{t}=F_{t}\cdot r,$$

where $T_{t}$: tangential torque [Nm], $F_{t}$: tangential force [N], r: radius of the wheel [m].

It is found that the maximum tangential torque developed by the athletes is $T_{t}=150\cdot 0.36=54\;Nm$. The study was conducted with athletes from the T54 class, meaning those with the lowest level of disability, i.e., participants who retain upper body muscular strength and may have muscular activity throughout the torso. In our study, the risk of adhesion loss occurs at torque values of at least 120 Nm. Thus, the risk of skidding seems unattainable.

One reason that could explain this result lies in the application of the torque to the wheel. Indeed, the skidometer was designed so that torque is applied gradually. However, in wheelchair sports, the torque applied to the hand rim is exerted abruptly. Therefore, it would be relevant to replicate the experiment to more closely reflect the real conditions on the field.

Moreover, we acknowledge that the formula $T_{t}=F_{t}\cdot r$ is based on the assumption of slip-free rolling, which may not hold true under skidding conditions. The hypothesis behind using this formula is to establish a baseline for comparison between the torques obtained in the experiment and those exerted by elite para-athletes under controlled conditions. While this model does not account for the complex dynamics of skidding, it provides a useful approximation for understanding the relative magnitudes of the forces involved. To address this limitation more comprehensively, future studies should consider more advanced biomechanical models that account for additional factors such as friction, deformation of the tire, and the abrupt application of torque. These factors introduce non-linearities and complex interactions that are not captured by the simple formula used.

Finally, during the experimentation, the treads of two tubulars (Dugast Pista Latex 20″ and 22″ pink) tore. As a result, they were not included in the Section 3. One possible explanation for this phenomenon lies in the tubulars’ tread material. It consists of a thin layer of latex directly bonded to the tubular tire to reduce rolling resistance. While this material is ideal for use on a cycling track, it appears unsuitable for use on an athletics track. The thin latex layer, while providing excellent performance on smooth surfaces, may not withstand the higher frictional forces and abrupt torque applications typical of athletics tracks.

Furthermore, it should be noted that the recommended pressure in the technical specifications ranges from 8 to 13 bars. In this protocol, the tested pressures ranged between 5.5 and 7 bars. This discrepancy in pressure settings could have contributed to the tread failure, as lower pressures may not provide sufficient support and stability for the tire under the demanding conditions of the experiment. By replicating the experiment under conditions that more closely reflect real-world scenarios, we can provide a more accurate representation of the forces and torques experienced by para-athletes during wheelchair racing, thereby enhancing the validity and applicability of our findings. This includes not only adjusting the pressure settings to align with the manufacturer’s recommendations but also considering the specific material properties and their suitability for different track surfaces. Future experiments should also explore a broader range of tubular materials and designs to identify those that offer the best balance of performance, durability, and safety for wheelchair athletes.

## 5. Conclusions

This study investigated the factors influencing wheel skidding in racing wheelchairs by measuring skidding torques using a skidometer. The parameters studied were the tubular type, the load applied to the wheel, the tire pressure, and the camber angle. The results demonstrated that tubular type and applied load significantly affect skidding torques. Indeed, higher loads reduce the risk of skidding and the Vittoria Pista Speed 23–28″ tubular was recommended to minimize the risk of skidding. Conversely, tire pressure and camber angle did not show significant effects within the tested ranges. These findings underscore the importance of selecting appropriate tubulars and considering athlete weight to minimize the risk of skidding. Future research should investigate a wider range of camber angles, examine the interactions between these factors, and consider rolling resistance as well as wet track conditions to gain a more comprehensive understanding of wheelchair racing dynamics. This work contributes to enhancing the performance and safety of para-athletes in competitive settings.

## Figures and Tables

**Table 1:** Summary of all factors studied.

**Type of tires (no. tires)** *	Michelin Power Cup Tubular Black (1); Dugast Strada 20″ black (2) and 22″ (3); Tufo Elite S3 (4); Vittoria Pista Speed 19–28″ (5) and 23–28″ (6)
Pressures [bars]	5.5; 6; 7
Load [kg]	20; 25; 30; 35
Camber [°]	11; 12

*: With tubulars having the following characteristics: (1) tubular with grip and a compound rubber mix; (2 and 3) tubular with grip and a neoprene coating; (4) tubular with grip and a rubber reinforcement; (5 and 6) smooth tire with a graphene coating.

**Table 2:** Taguchi L16 table used for each type of tubular. Each row represents a tested combination. Each number indicates a factor level (see Table 3).

	Tire	Pressure	Load	Camber
1	x *	1	1	1
2	x	2	2	2
3	x	3	3	1
4	x	1	4	2
5	x	1	2	1
6	x	2	1	2
7	x	3	4	1
8	x	3	3	2
9	x	1	3	2
10	x	2	4	1
11	x	3	1	2
12	x	2	2	1
13	x	1	1	2
14	x	2	3	1
15	x	3	2	2
16	x	2	1	1

*: The level of the tire factor is denoted by the letter x, as this table is identical for each tubular type, with x varying from 1 to 6 based on the specific tubular type under investigation.

**Table 3:** Factor level values are associated with the Taguchi table.

Factor Level	Associated Pressure [bars]	Associated Load [kg]	Associated Camber [°]
1	5.5	20	11
2	6	25	12
3	7	30	N.V
4	N.V *	35	N.V

*: The indication N.V stands for “no value”. This means that there are no tested values for this factor level. The parameters do not have an identical number of factors studied.

**Table 4:** Results of the multiple one-way ANOVAs. The factors are tubular type, load, pressure, and camber.

Parameters	*F* *	*p*	*η* ^2^	*F*	*p*	*η* ^2^
	Grip Loss			Grip Recovery		
Tubular	10.3	<0.001 ***	0.250	11.47	<0.001 ***	0.270
Load	157.5	<0.001 ***	0.778	130.5	<0.001 ***	0.744
Pressure	/	NS	/	/	NS	/
Camber	/	NS	/	/	NS	/

* with F: ANOVA result; p: *p*-value set at 0.05; $\eta^{2}$: effect size for the significant difference in the ANOVA. Significance levels of the ANOVA tests: * low (*p* < 0.05), *** high (*p* < 0.001).

## Data Availability

Data are available upon reasonable request from the corresponding author.
